# Seed Treatment with Diamide and Neonicotinoid Mixtures for Controlling Fall Armyworm on Corn: Toxicity Evaluation, Effects on Plant Growth and Residuality

**DOI:** 10.3389/fchem.2022.925171

**Published:** 2022-06-08

**Authors:** Hongbo Li, Lei Feng, Junhong Fu, Ying Zhang, Wenyuan Huang, Tingting Duan, Yang Hu, Jichun Xing

**Affiliations:** ^1^ Institute of Plant Protection, Guizhou Academy of Agricultural Sciences, Guiyang, China; ^2^ Guizhou Provincial Key Laboratory for Agricultural Pest Management of the Mountainous Region, School of Agriculture, Institute of Entomology, Guizhou University, Guiyang, China

**Keywords:** *Spodoptera frugiperda*, diamide, neonicotinoid, seed treatment, combined toxicity

## Abstract

The diamides, chlorantraniliprole (CHL) and cyantraniliprole (CYA), have been used as seed treatment agents against the fall armyworm (FAW), *Spodoptera frugiperda* in China. However, large-scale application of these two insecticides is prohibited because of their high cost. The neonicotinoid insecticides, clothianidin (CLO) and thiamethoxam (THI), are cheaper and widely used. In this study, we tested the efficacy of CHL + CLO and CYA + THI as seed treatment agents against FAW larvae both in laboratory and field conditions. Laboratory experiments showed that the two binary mixtures (both 240 g.a.i.100 kg^−1^ corn seeds) caused FAW mortality exceeded 84.00% at 14 days after seedling emergence (DAE). The mortality of the binary mixtures were similar to either CHL (300 g.a.i.100 kg^−1^corn seeds) or CYA (144 g a.i.100 kg^−1^corn seeds), but higher than CLO (120 g.a.i.100 Kg^−1^corn seeds) or THI (180 g a.i.100 kg^−1^corn seeds). Two independent field experiments showed that both binary mixtures resulted in above 68.00% control efficacy at 14 DAE, suggesting that these insecticidal combinations could effectively control FAW over a relative long period. In addition, both binary mixtures showed no negative effects on the growth and development of corn seedlings. The residues of binary mixtures in corn leave were also lower at 28 DAE as compared to residues in CHL or CYA alone. Most importantly, the costs of CHL + CLO were reduced up to 50% and CYA + THI up to 20% when compared to singly used chemical. Totally, our results indicated that CHL + CLO and CYA + THI had the same control efficacy as CHL or CYA alone, but with much lower cost.

## Introduction

Corn is a globally-important food crop, that is, consumed by approximately 4.5 billion people worldwide (Shiferaw et al., 2011). In the field, corn yield is reduced by multiple insect pests, including *Spodoptera frugiperda* Smith (Lepidoptera: Noctuidae)*. S. frugiperda*, also known as the Fall Armyworm (FAW), is a native corn pest in tropical and subtropical areas of North America (Martinelli et al., 2006). In recent years, the FAW has invaded Africa and Asia. This pest is highly destructive due to its wide host range, robust migration ability, high fecundity and resistance to insecticides (Guo et al., 2019). In China, FAW was first found in Yunnan province in December 2018 (Sun et al., 2021); it quickly spread throughout 26 provinces and damaged approximately 65.53 × 10^4^ hm^2^ corn (Jiang et al., 2019). The FAW is now well-established in winter corn grown in southern China and is a dominant corn pest due to its migratory ability (Qi et al., 2020; Jiang et al., 2021).

Spraying chemical insecticides remains as the most effective measure for controlling this pest. However, there are several factors reducing the efficacy of insecticide spraying against FAW. For example, the efficacy of insecticidal sprays is largely effected by weather and subject to dilution by rain and wind (Zheng et al., 2006; Ranabhat and Wang, 2020). The efficacy of insecticides is also impacted by larval behavior; this is especially relevant for FAW since larvae hide inside the maize whorl, which reduces their exposure to insecticides (Muraro et al., 2020). A further barrier is the labor shortage, which can delay spray applications and reduce efficacy. Finally, the overuse of chemical insecticides contaminates the environment and has impacts on mammals and nontarget arthropods (Lahm et al., 2007).

Seed treatment with systemic insecticides has been used to control pests on many crops (Taylor et al., 2001). In general, treating seeds with chemicals requires less insecticide than spray application and reduces environmental contamination and exposure of nontarget organisms (Schemeer et al., 1990; Nault et al., 2004). Consequently, seed treatment is popular in integrated pest management programs (Zhang et al., 2011). Previous studies demonstrated that seed treatment with carbofuran or thiamethoxam (THI) was ineffective for controlling early larval stages of FAW on corn (Azevedo et al., 2004). Chlorantraniliprole (CHL) and cyantraniliprole (CYA) are anthranilic diamides that target insect ryanodine receptors and disrupt the functioning of calcium channels (Lahm et al., 2007). These two compounds control insect pests by directly killing individuals and inhibiting their feeding, development and reproduction (Huang et al., 2016; Lutz et al., 2018). Previous studies demonstrated that these insecticides showed excellent control efficacy, particularly when used for lepidopteran pests such as FAW (Wang et al., 2019a; Pes et al., 2020). Recently, CHL and CYA were labeled as seed treatments for the control of FAW larvae (Muraro et al., 2020; Pes et al., 2020) because of their low LogPow (octanol/water partition coefficient) and high solubility in water (Selby et al., 2017).; unfortunately, the cost of CHL and CYA is much more expensive than traditional insecticides.

The combined application of two different insecticides could improve control of target pests (Thrash et al., 2013; Carscallen et al., 2019; Muraro et al., 2020). The dual application of anthranilic diamides with other insecticides for FAW control is under-investigated but clearly needed to reduce the cost of control. Thus, the objective of this study was to compare the efficacy of corn seed treatments with CHL, CYA, clothianidin (CLO), THI and the binary mixture of CHL + CLO and CYA + THI for control of FAW larvae in laboratory and field conditions. A recent study suggested that the application of selected insecticides as seed treatments inhibited crop growth because of prolonged, high residue level (Abdu-Allah and Hashem, 2017). Thus, we also evaluated the effects of the above-mentioned chemicals on growth of corn plants and determined insecticide residue levels.

## Materials and Methods

### Insects

FAW populations for laboratory experiments were collected in 2019 from corn fields located at the Guizhou Academy of Agricultural Sciences in Guizhou province, China. The larvae were reared on a corn-based artificial dietat 25 ± 1°C and 70% relative humidity (RH) with a 16:8 h (L:D) photoperiod (Wang et al., 2019b). Third instar larvae were used in the laboratory experiments.

### Corn Seeds and Insecticides

Corn seeds of the cultivar Jinyu 818 were provided by Guizhou Jinlong Technology Co., Ltd. Insecticides were sourced from the following companies: chlorantraniliprole (CHL, 50% FSC), DuPont Crop Protection (United States); cyantraniliprole + thiamethoxam (CYA + THI, 60% FSC) and cyantraniliprole (CYA, 40% FSC), Syngenta AG (Switzerland); chlorantraniliprole + clothianidin (CHL + CLO, 40% FSC), Guangdong Kairuifeng Technology Co., Ltd. (China); clothianidin (CLO, 20% FSC), Hebei Lishijie Technology Co. (China); and thiamethoxam (THI, 30% FSC), Bayer Crop Science LP, Monheim (Germany).

### Laboratory Experiments

Laboratory experiments were conducted from May to July in 2021 and consisted of the following seven treatments (concentrations in g a.i.100 kg^−1^corn seeds): 1) CHL, 300; 2) CYA, 144; 3) CHL 60 + CLO 180; 4) CYA 120 + THI 120; 5) CLO, 120; 6) THI, 180; and 7) untreated control. The concentration of each insecticide was chosen based on recommended field rates.

One day prior to sowing, seeds and pesticides were placed in plastic bags, sealed, shaken until insecticides coated the seed surface, and allowed to dry overnight. Fifty corn seeds were sown in individual containers (30 × 20 × 20 cm) containing sand (40%), clay (40%) and organic matter (20%) at 25°C, 70% RH and a 14:10 (L:D) photoperiod. Water was provided during seed emergence and growth as necessary, and the emergence rate of seeds in each treatment was recorded. At 3, 7, 14, 21, 28 days after seedling emergence (DAE), twenty newly-molted 3rd instar larvae were collected, starved for 2 h, and then transferred to corn plants. Finally, the plants together with FAW larvae were placed in nylon cages to prevent escaping. Each potting container was considered as one replication, and each treatment had four replications. Larval mortality was recorded after 3 days transferring, and larvae were considered dead if there was no response to stimulation with a moist brush.

### Field Experiments

Two identical field experiments were conducted to evaluate the efficacy of six insecticidal formulations for FAW larval control on corn seedlings in Luodian county (106.63^°^E, 25.62^°^N), Guizhou province, in July and September of 2021. In the two field experiments, 28 plots were arranged in a randomized complete block design with seven treatments and four replications. Insecticide-treated corn seeds were sown on 23 June and 23 August 2021. Each plot was 30 m^2^ (5 × 6 m) and consisted of 10 rows separated by 60 cm of uncultivated ground. The emergence rate of corn seeds was recorded by counting the number of emerged plants in each plot. A five-point sampling method where each point consisted of 10 plants was used to record the number of FAW larvae on corn in each plot at 7, 14, 21, and 28 DAE. The damage rate caused by FAW and the control efficacy of tested insecticides were calculated by the following formulas:

$$\mathrm{Percentage}\;\mathrm{of}\;\mathrm{corn}\;\mathrm{seedlings}\;\mathrm{with}\;\mathrm{FAW}\;\mathrm{damage}\;\left(\%\right)=\frac{\mathrm{\#corn}\;\mathrm{plant}\;\mathrm{with}\;\mathrm{FAW}\;\mathrm{damage}}{\mathrm{\#investigated}\;\mathrm{corn}\;\mathrm{plants}}\mathrm{\times 100\%}$$ (1)

$$\mathrm{Control}\;\mathrm{efficacy}\left(\%\right)=\frac{\mathrm{\#FAW}\;\mathrm{in}\;\mathrm{untreated}\;\mathrm{plot- \#FAW}\;\mathrm{in}\;\mathrm{treated}\;\mathrm{plot}}{\mathrm{\#FAW}\;\mathrm{in}\;\mathrm{treated}\;\mathrm{plot}}\mathrm{\times 100\%}$$ (2)

### Effect of Insecticides on Fall Armyworm Emergence and Corn Growth

The emergence rate of corn seeds treated with the six insecticidal formulations were recorded at 7 DAE in both laboratory and field experiments. The impact of the six insecticides on corn grown was evaluated by random selection of 20 plants in each plot at 14 DAE. Plant height, root length, and above-ground and underground fresh weight were measured in July and September of 2021.

### Determination of Residual Insecticides in Corn Leaves

Corn leaves were randomly selected from field plots at 3, 7, 14, 21, and 28 DAE in July 2021 and stored at −20°C until needed for analysis. Homogenized corn leaves (5.0 ± 0.1 g) were weighed in 50 ml Teflon centrifuge tubes, and water (5 ml) and 10 ml acetonitrile with 1% (*v/v*) methanol (HOAc) were added to samples. Sample tubes were shaken vigorously for 10 min, allowed to stand for 30 min, and NaCl (3 g) and MgSO_4_ (4 g) were added. Tubes were capped, mixed for 1 min and centrifuged for 5 min at 3216 × g. The upper acetonitrile layer (1.5 ml) was transferred into 2.0 ml tubes containing 50 mg octadecylsilane (C_18_), 15 mg graphitized carbon black (GCB) and 150 mg MgSO_4_. The tubes were vortexed for 30 s and centrifuged for 5 min at 2233 × g. The resulting supernatants were subjected to ultrafiltration (0.22 μm nylon filter) and then loaded into auto-sampler vials for UHPLC-MS/MS analysis.

Chromatographic separation of CLO, THI, CHL, CYA, and J9Z38 (metabolite of CYA) was performed using a Dionex Ultimate 3000 UHPLC system and a Syncronis C_18_ column (100 mm × 2.1 mm, 1.9 µm) (Thermo Fisher Scientific, United States) at 40°C with a 5 µl injection volume. The mobile phases consisted of solution A (H_2_O containing 0.1% *v/v* formic acid) and solution B (methanol); the flow rate was 0.25 ml·min^−1^. The elution program was: 25% solvent B from 0 to 1.0 min; 25%–85% solvent B from 1.0 to 1.5 min; 85% solvent B from 1.5 to 6 min; 85%–25% solvent B from 6 to 6.5 min; and 25% solvent B for 1.5 min. Qualitative and quantitative analysis of CLO, THI, CHL, CYA, and J9Z38 were obtained with a triple-quadrupole mass spectrometer (TSQ Vantage) equipped with an ESI interface (Thermo, San Jose, CA, United States). Nitrogen was used as the sheath and auxiliary gas at 30 and 10 PSI, respectively. The vaporizer and capillary temperatures were both 330°C, and the spray voltage was 3.2 kV. The pressure of argon in the collision cell was 1.5 mTorr. The MS/MS conditions were optimized to acquire satisfactory sensitivity and resolution using the parameters listed in Supplementary Table S1. First-order kinetic and bi-exponential models were used to analyze the dissipation curves of CLO, THI, CHL, CYA, and J9Z38. Analysis of t kinetics was performed in KinGUIIv2.1 (BASF Corporation) as follows:

$$C\;=\;C_{0}e^{-kt}$$ (3)

$$T_{1/2}\;=\;ln2/K$$ (4)

### Statistical Analysis

Data associated with mortality were arcsine-transformed before statistical analysis. One-way ANOVA was used to determine statistical significance among treatments, followed by a Tukey’s HSD method. Results were considered significant at *p* < 0.05. Statistical analyses were performed in DPS v. 17.0 (Tang and Zhang, 2013).

## Results

### Efficacy of Insecticidal Seed Treatments in Laboratory Experiments

**FIGURE 1:**
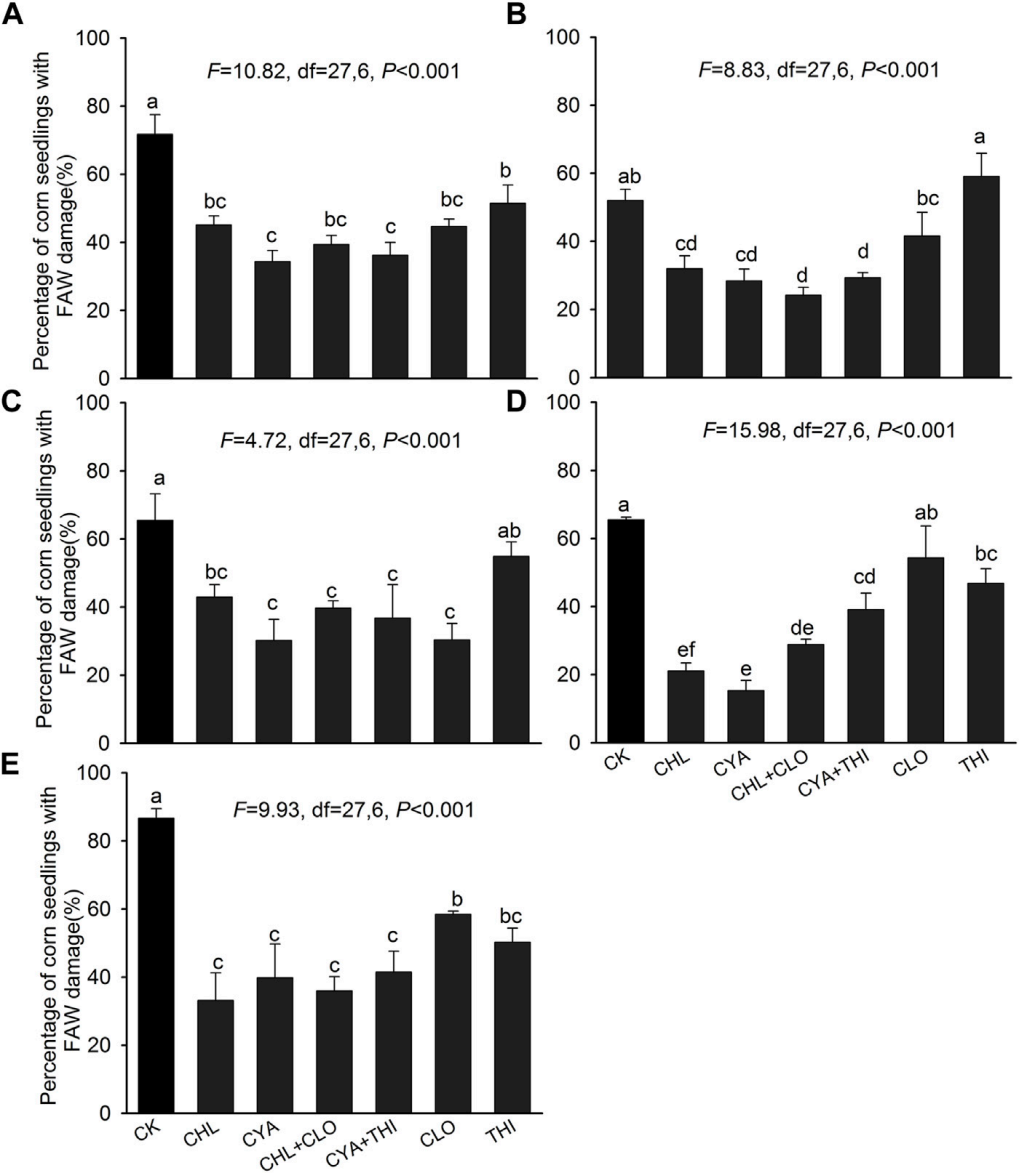
Percentage of corn seedlings damaged by FAW in laboratory experiments corn where seeds were treated with chlorantraniliprole (CHL), cyantraniliprole (CYA), clothianidin (CLO), thiamethoxam (THI), CHL + CLO and CYA + THI; CK is the untreated control. Damage is shown on corn seedlings subjected to FAW larvae at 3 panel **(A)**, 7 **(B)**, 14 **(C)**, 21 **(D)**, and 28 **(E)** days after emergence (DAE). Columns labeled with different letters indicate significant differences among treatments with Tukey’s HSD test at *p* < 0.05.

In laboratory experiments, the percentage of corn seedlings damaged by FAW in CHL + CLO, CYA + THI, CHL, and CYA ranged from 15.35 to 45.13% at 3–28 DAE, and these levels were significantly lower than the untreated control where damage was 52.03%–86.70% (Figure 1). The percentage of corn seedlings with FAW damage in CLO or THI alone was generally either slightly lower or not significantly different from the untreated control (Figures 1A–E).

The corrected mortality of FAW larvae fed on corn plants treated with the six insecticides declined as days after seed emergence increased (Table 1). At DAE 3, 7, and 14, CHL, CYA, CHL + CLO and CYA + THI treatments resulted in similar levels of mortality and ranged from 72.00% to 94.44%; furthermore, FAW mortality in these four treatments was significantly higher than in larvae exposed to CLO and THI treatments. At 21 or 28 DAE, mortality in the CHL, CYA, CHL + CLO and CYA + THI treatments ranged from 60.00% to 72.97% and remained significantly higher than mortality in the CLO or THI treatments.

**TABLE 1:** The corrected mortality (±SE) of FAW larvae fed on corn plants subjected to insecticidal seed treatments in laboratory experiments.

Treatments	Dosage (ga.i.100 kg^−1^seeds)	Corrected mortality (%)				
		3 DAE	7 DAE	14 DAE	21 DAE	28 DAE
CHL	300	88.89 ± 2.27a	78.08 ± 2.24b	72.00 ± 1.33b	63.51 ± 1.35a	61.43 ± 2.74a
CYA	144	90.28 ± 3.21a	89.04 ± 2.24a	78.67 ± 5.33ab	66.22 ± 1.35a	60.00 ± 6.17a
CHL + CLO	240	94.44 ± 1.39a	90.41 ± 2.62a	89.33 ± 4.87a	71.62 ± 2.59a	64.29 ± 5.89a
CYA + TH	240	94.44 ± 2.27a	93.15 ± 1.37a	84.00 ± 2.18ab	72.97 ± 6.62a	61.43 ± 2.74a
CLO	120	65.28 ± 4.74b	58.90 ± 1.58c	54.67 ± 1.54c	35.14 ± 6.24b	17.14 ± 1.65b
THI	180	58.33 ± 3.59b	43.84 ± 4.11d	41.33 ± 3.77d	40.54 ± 2.21b	18.57 ± 1.43b
*F*	-	14.12	50.47	14.14	14.84	25.72
df	-	23.5	23.5	23.5	23.5	23.5
*p*	-	<0.001	<0.001	<0.001	<0.001	<0.001

Values followed by different letters indicate significant differences among treatments with Tukey’s HSD test at *p* < 0.05.

### Efficacy of Insecticidal Seed Treatments in Field Experiments

**FIGURE 2:**
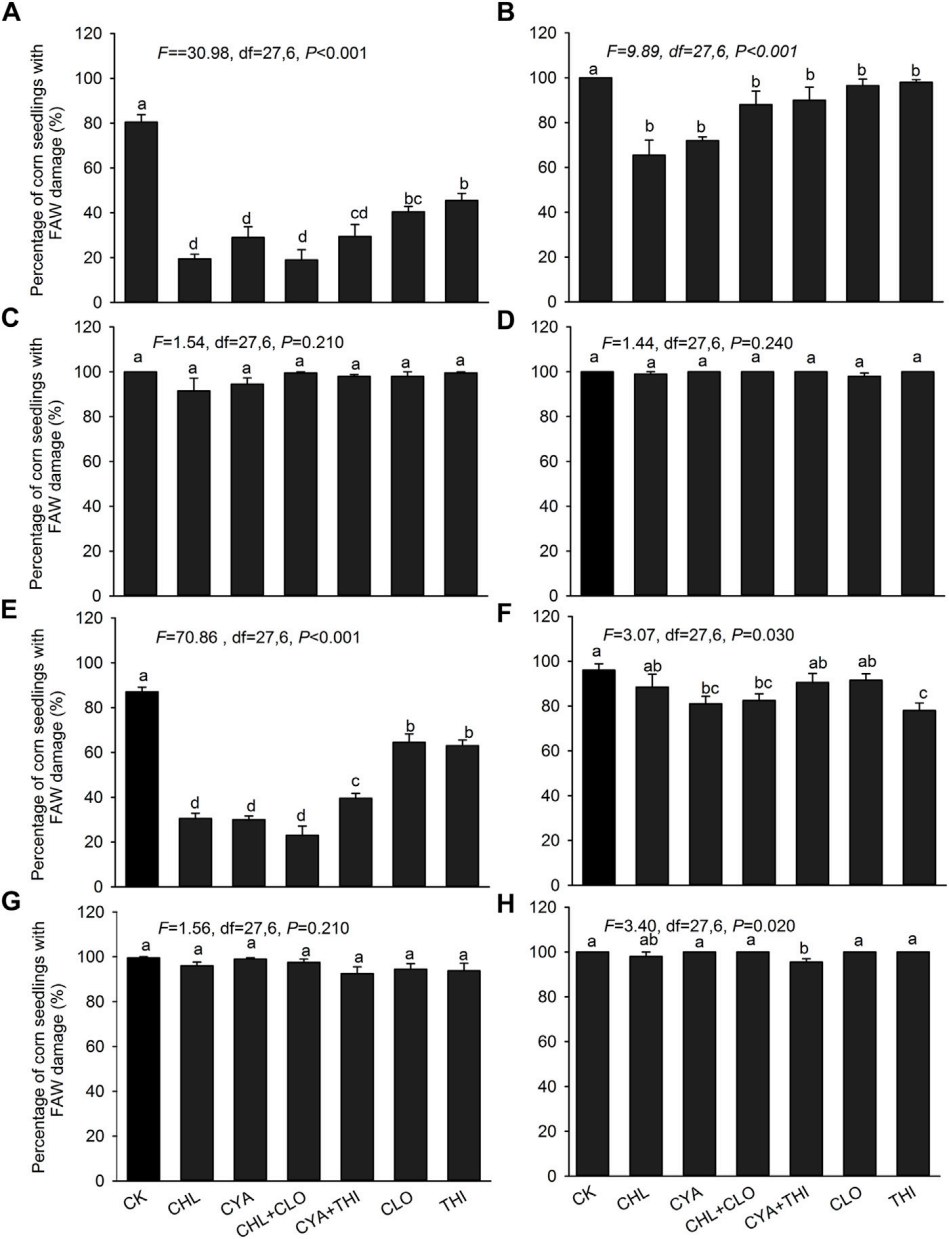
Percentage of corn seedlings damaged by FAW in field experiments corn where seeds were treated with chlorantraniliprole (CHL), cyantraniliprole (CYA), clothianidin (CLO), thiamethoxam (THI), CHL + CLO and CYA + THI; CK is the untreated control. Damage rates (±SE) caused by FAW larvae are shown for July 2021 at 7 panel**(A)**, 14 **(B)**, 21 **(C)** and 28 **(D)** DAE. Damage rates in September 2021 are shown for 7 panel **(E)**, 14 **(F)**, 21 **(G)** and 28 **(H)** DAE. Columns labeled with different letters indicate significant differences among treatments with Tukey’s HSD test at *p* < 0.05.

In July 2021, the percentage of corn seedlings damaged by FAW in insecticide-treated field plots ranged from 19.50% to 63.00% at 7 DAE, which was significantly lower than the control (80.00%) (Figure 2A). At 14 DAE, the value in insecticide-treated plots showed a substantial increase but were still significantly lower than the untreated control (Figure 2B). At 21 and 28 DAE, the value in treated plots were not significantly different from the control (Figures 2C,D). The percentage of corn seedlings with FAW damage in September 2021 at all four sampling times were similar to results in July (Figures 2E–H).

In July, CHL, CYA, CHL + CLO and CYA + THI treatments resulted in 79.84%–87.88% control efficacy at 7 DAE, which were significantly higher than those in the CLO and THI treatments (Table 2). Furthermore, control efficacy in the CYA + THI treatment was higher than in corn treated with CYA alone. At 14 DAE, control efficacy in the CHL, CYA, CHL + CLO and CYA + THI treatments were higher than CLO and THI treatments; however, the efficacy of the CHL, CYA, CHL + CLO and CYA + THI treatments decreased dramatically at 21 and 28 DAE (Table 2). In September, CHL, CYA, CHL + CLO and CYA + THI treatments resulted in control efficacy ranging from 84.43% to 87.73% and 68.33%–74.79% at 7 and 14 DAE, respectively, and the values were significantly higher than those in the CLO and THI treatments. Similar to results obtained in July, the efficacy in CHL, CYA, CHL + CLO and CYA + THI treatments decreased at 21 and 28 DAE and were not significantly different from the CLO and THI treatments (Table 2).

**TABLE 2:** Control efficacy (mean ± SE) of six insecticidal seed treatments for control of FAW on corn in the field.

Experimental time	Treatments	Dosage (ga.i.100 kg^−1^seeds)	Control efficacy (%)			
			7 DAE	14 DAE	21 DAE	28 DAE
July	CHL	300	84.15 ± 2.82ab	76.42 ± 2.22ab	55.79 ± 4.37ab	40.94 ± 2.45b
	CYA	144	79.84 ± 3.45b	68.93 ± 3.82b	49.54 ± 4.76bc	14.09 ± 7.02d
	CHL + CLO	240	87.88 ± 1.83a	77.94 ± 3.25a	48.61 ± 1.71bc	32.21 ± 4.57b
	CYA + THI	240	86.71 ± 1.92a	71.46 + 1.51ab	44.68 ± 2.34c	19.46 ± 1.10cd
	CLO	120	65.97 ± 2.20c	57.09 ± 4.14d	57.87 ± 1.39a	31.54 ± 4.17bc
	THI	180	72.49 ± 2.20c	62.35 ± 1.99cd	58.33 ± 2.17a	53.69 ± 2.29a
	*F*	-	14.68	7.27	3.36	12.34
	df	-	23.5	23.5	23.5	23.5
	*p*	-	<0.001	<0.001	0.03	<0.001
September	CHL	300	84.43 ± 1.09a	74.79 ± 3.12a	61.23 ± 5.64ab	66.79 ± 4.20a
	CYA	144	87.00 ± 1.92a	70.15 ± 1.67a	62.28 ± 2.90a	63.38 ± 1.78a
	CHL + CLO	240	85.53 ± 1.85a	68.33 ± 0.74a	57.56 ± 2.91ab	66.43 ± 3.46a
	CYA + THI	240	87.73 ± 0.92a	72.31 ± 1.06a	50.00 ± 5.56b	64.63 ± 1.56a
	CLO	120	41.94 ± 3.63b	58.54 ± 2.79b	51.02 ± 2.13ab	59.43 ± 3.60a
	THI	180	42.67 ± 2.21b	61.19 ± 2.43b	57.8 ± 2.51ab	62.84 ± 2.61a
	*F*	-	113.42	8.77	3.73	0.80
	df	-	23.5	23.5	23.5	23.5
	*p*	-	<0.001	<0.001	0.02	0.56

Values with different letters indicate significant differences with Tukey’s HSD test at *p* < 0.05.

### Effect of Insecticides on Emergence Rates and Corn Growth

**FIGURE 3:**
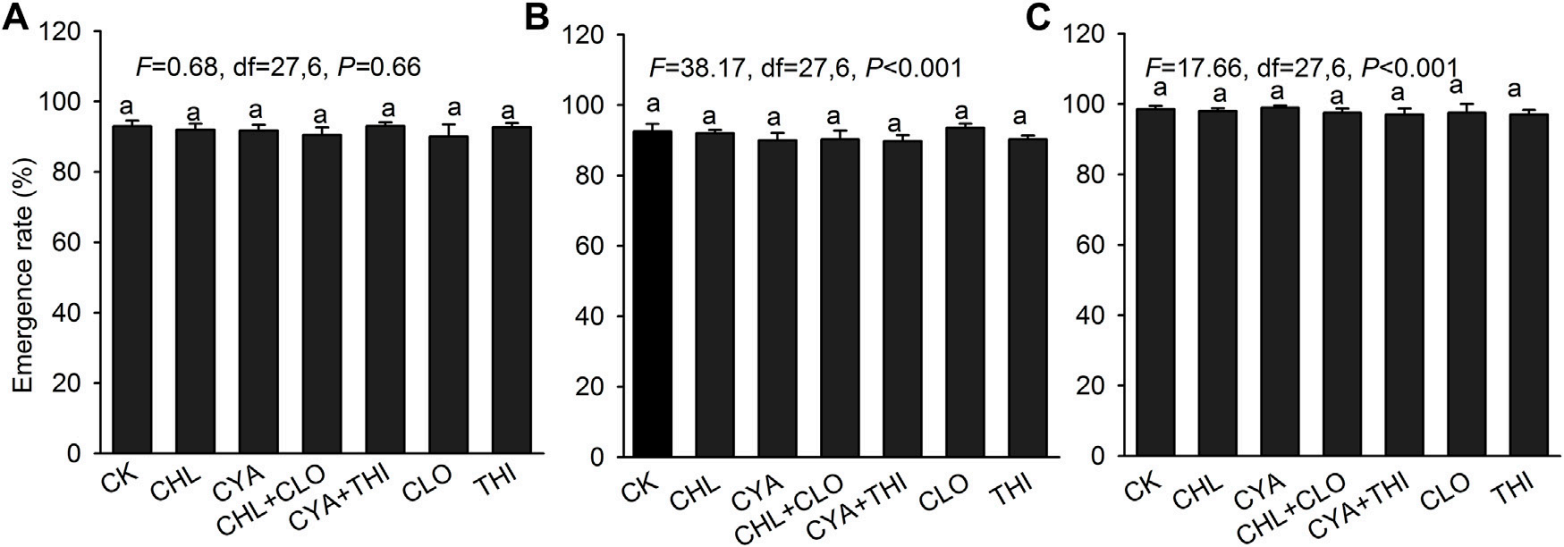
The emergence rate (±SE) of corn seeds after treatment with chlorantraniliprole (CHL), cyantraniliprole (CYA), clothianidin (CLO), thiamethoxam (THI), CHL + CLO and CYA + THI; CK is the untreated control. Panels: **(A)** emergence rate of corn seeds in laboratory experiments; **(B)** and **(C)** emergence rate of corn seeds in field experiments in July and September 2021, respectively. Columns labeled with the same letter were not significantly different using Tukey’s HSD test at *p* < 0.05.

**FIGURE 4:**
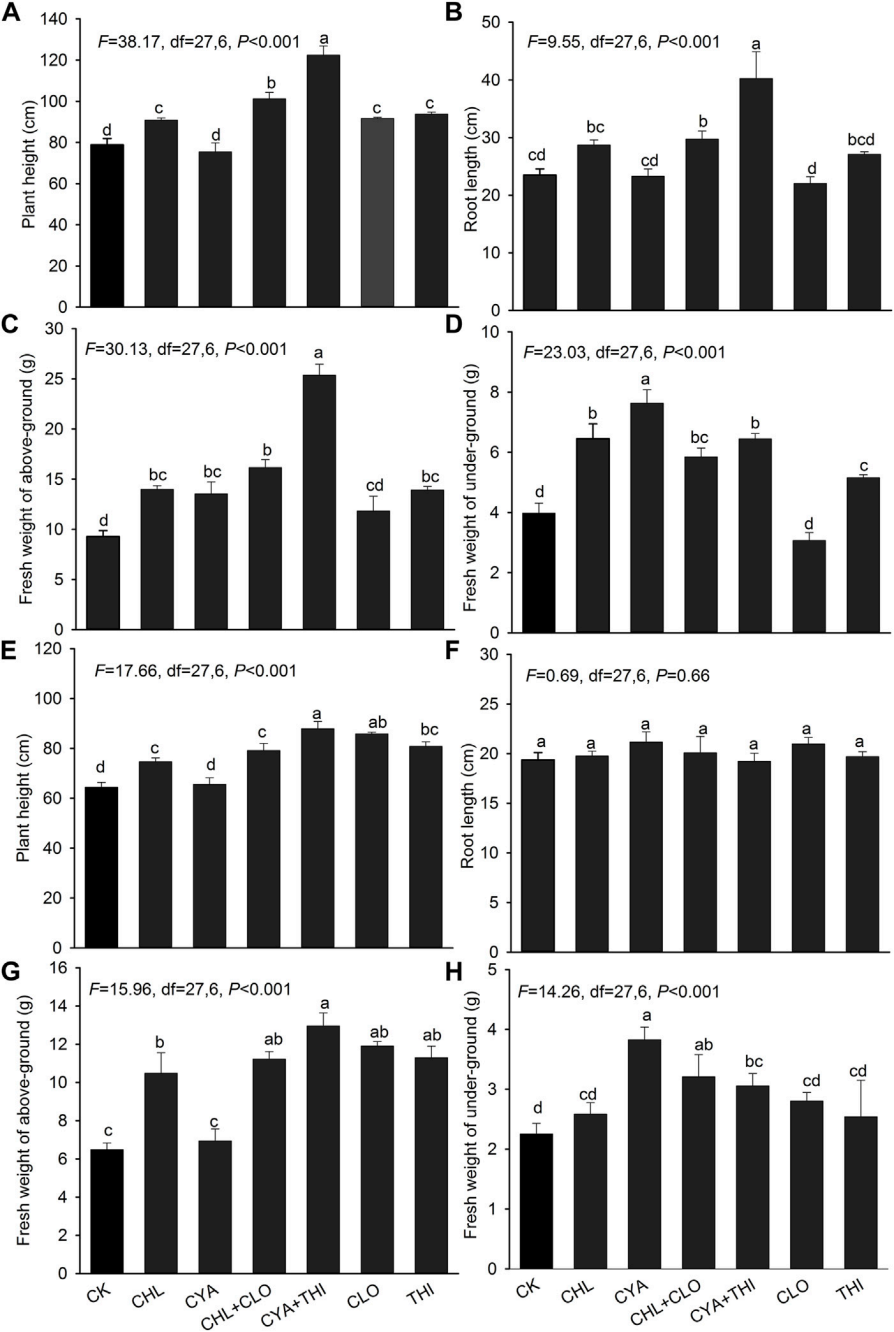
The mean plant height, root length, and fresh weight above-ground and underground of corn seedlings treated with CHL, CYA, CLO, THI, CHL + CLO and CYA + THI in the July **(A–D)** and September **(E–H)** of 2021. Columns labeled with the same letter were not significantly different using Tukey’s HSD test at *p* < 0.05.

The emergence rate of corn treated with the six different insecticide formulations exceeded 90% in both laboratory and field experiments with no significant differences between treatments (Figure 3). In July 2021, corn seedlings in field plots treated with CHL + CLO and CYA + THI were significantly taller than seedlings in other treatments (Figure 4A). Root length and fresh weight of above-ground tissue were generally higher in seedlings treated with CHL + CLO and CYA + THI as compared to the other four treatments, although these differences were not always significant (Figures 4B,C). The underground fresh weights in CHL + CLO and CYA + THI treatments were significantly higher than those in CLO treatment and control (Figure 4D). In September, data points for plant height and fresh weight of above-ground and underground tissues were similar to those recorded in July for all treatments (Figures 4E,G,H). Unlike July, the root length data in September was not significantly different among treatments (Figure 4F).

### Insecticide Residues in Treated Corn Plants

**FIGURE 5:**
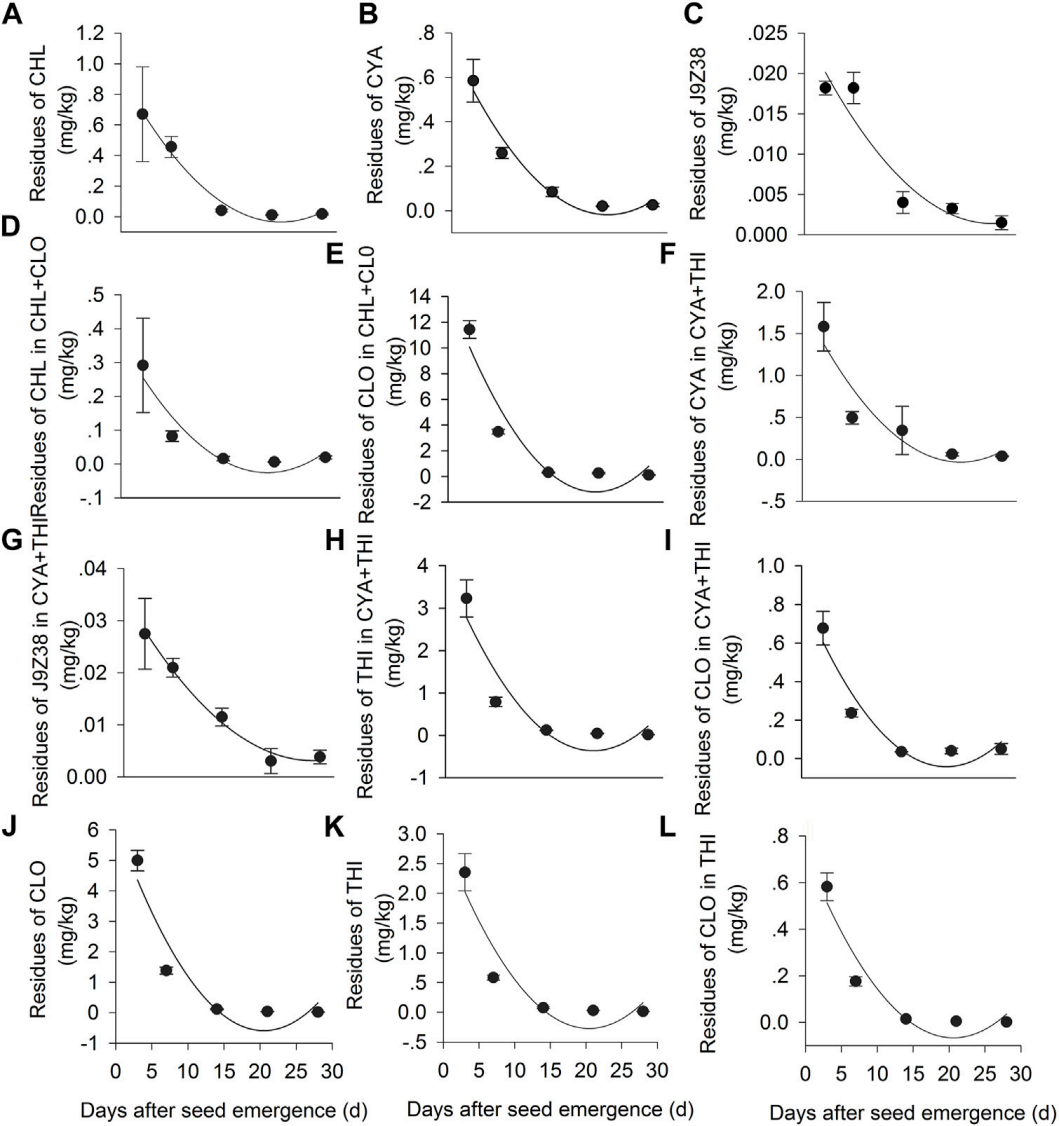
Dynamic changes in the concentrations of insecticides and their metabolites in CHL, CYA, CHL + CLO, CYA + THI, CLO, THI treated leaves in July corn plants. Panels: **(A)** CHL residue in CHL treated leaves; **(B)** CYA residues in CYA treated leaves; **(C)** J9Z38 residues in CYA treatedleaves; **(D)** CHL residues in CHL + CLO treated leaves; **(E)** CLO residues in CHL + CLO treatedleaves; **(F)** CYA residues in CYA + THI treated leaves; **(G)** J9Z38 residues in CYA + THI treatedleaves; **(H)** THI residues in CYA + THI treated leaves; **(I)** CLO residue in CYA + THI treated leaves; **(J)** CLO residue in CLO treated leaves; **(K)** THI residues in THI treated leaves; **(L)** CLO residues in THI treated leaves. Fitted regression lines are from the equation C_t_ = C_0_e^−bt^, and showed in Supplementary Table S2.

Residues levels of the six insecticidal formulations and their metabolites gradually decreased in corn plants throughout the sampling period (Figure 5). For example, in the CHL + CLO treatment, the CHL residues declined from 0.29 mg·kg^−1^ at 3 DAE to 0.02 mg·kg^−1^ at 28 DAE, while the CLO residues declined from 11.44 mg·kg^−1^ at 3 DAE to 0.12 at 28 DAE (Figure 5E). The half-life (t_1/2_) of CHL in corn plants treated with CHL + CLO was 2.32 days, which was shorter than that in the CHL treatment alone (4.35 days) (Supplementary Table S2). The half-life of CLO in CHL + CLO treated corn plants was 2.26 days, which was similar to that in the CLO treatment alone (2.15 days) (Figure 5A, Supplementary Table S2).

In the CYA + THI treatment, residues of CYA and its metabolite J9Z38 degraded from 1.58 to 0.04 mg·kg^−1^ and 0.03–0.00 mg·kg^−1^, respectively, during 3–28 DAE period (Figures 5F,G). Residues of THI and its metabolite (CLO) degraded from 3.23 to 0.02 mg·kg^−1^ and 0.68 to 0.03 mg·kg^−1^, respectively, during the same period (Figures 5H,I). The estimated half-life of the CYA, J9Z38, THI and CLO in CYA + THI combination were 3.07, 7.76, 1.99 and 2.67 days, respectively, most of which were low when compared to the CYA or THI treatment alone (Figures 5B,C,K,L, Supplementary Table S2).

### Comparison of the Cost of the Insecticides

As shown in Supplementary Table S3, the cost of both CHL + CLO and CYA + THI were below 50.00 $ ha^−1^, which lead to reduced cost of 42.52 $ ha^−1^ and 11.34 $ ha^−1^ when compared to CHL (85.04 $ ha^−1^) and CYA (56.69 $ ha^−1^), respectively.

## Discussion

The diamide insecticides kill insect pests by targeting their ryanodine receptor channels (RyRs) that cause muscle contraction and death (Lahm et al., 2007), while the neonicotinoid insecticides kill insect pests by targeting their nicotinic acetylcholine receptors (nAChRs) (Matsuda et al., 2020). In previous laboratory studies, the diamide insecticides such as CHL and CYA were highly toxic to FAW larvae with LC_50_ values of 0.01 and 0.25 μg ml^−1^, respectively (Bolzan et al., 2019; Zhou et al., 2020). These two insecticides were recently labeled as a seed treatment for controlling FAW and exhibited a high level of control efficacy against this species (Zhang P. et al., 2016; Pes et al., 2020). Neonicotinoid insecticides such as CLO and THI have been widely used as seed treatments in multiple crops and exhibit good activity against a broad range of pests including aphids (Zhang P. et al., 2016; Zhang Z. et al., 2016), whiteflies (Zhang et al., 2011), and thrips (Reisig et al., 2012; Ding et al., 2018). To the best of our knowledge, there are no prior studies assessing the efficacy of diamides in combination with neonicotinoids for FAW larval control as seed treatments.

CHL + CLO, CYA + THI, CHL, and CYA were effective at reducing percentage of corn seedlings with FAW damage in the laboratory. The percentage of corn seedlings with FAW damage was similar among the four treatments, suggesting that CHL + CLO and CYA + THI were comparable to CHL and CYA treatment alone. Prior studies reported that CHL can rapidly inhibit the feeding of some lepidopteran pests. For example, the feeding behavior of *Trichoplusia ni*, *Plutella xylostella* and *Helicoverpa zea* ended within 30 min after exposure to CHL, and damage decreased by 90%–99% (Hannig et al., 2009). Therefore, diamides alone or in combination with neonicotinoid insecticides reduce damage by causing early mortality, inhibiting feeding, and disrupting larval development, which is similar to results reported elsewhere (Hannig et al., 2009; Carscallen et al., 2019).

The percentage of corn seedlings with FAW damage in our two field experiments was generally higher than in the laboratory, which may be attributed to one or more of the following reasons, Firstly, 3rd instar larvae were used in the lab studies, and this life stage is relatively stable and uniform. Secondly, the high damage rate in the field is likely related to FAW behavior and environmental conditions; for example, FAW adults randomly deposit eggs on corn and the newly-hatched larvae are disseminated by wind, thus increasing range and the damage rate of corn. Therefore, damage rates may not be a suitable index for evaluating the efficacy of insecticides when applied as seed treatments in the field. Recently, an injury score rating was used to evaluate the control efficacy of seed coatings by measuring the feeding area in some lepidopteran species (Carscallen et al., 2019; Wu et al., 2020). It is unclear whether this index is suitable for FAW and further study is needed.

Our lab experiments showed that application of CHL + CLO and CYA + THI to corn resulted in high FAW mortality (84.00%–94.44%) at 14 DAE, which indicates that the combined application of insecticides was an effective control strategy. Our results were similar to those reported for application of CHL and CYA alone in one study (Wu et al., 2020), but were much higher than results reported by Thrash et al. (2013). These disparate outcomes may be caused by variability in plant hosts, FAW populations and insecticide doses. The effectiveness of diamide insecticides alone or in combination with neonicotinoids has also been reported for *Mythimna unipuncta* (Carscallen et al., 2019). Furthermore, as corn plants grew larger in the present study, the corrected mortality of FAW larvae in all treatments decreased; this is likely due to the decline in insecticidal residues and increase in body size of larvae over time. We also observed that the CLO and THI treatments caused mortality from 41% to 65.28% at 14 DAE, which indicates that these two insecticides are somewhat effective in controlling FAW larvae. These data are consistent with previous results reported for *Agrotis ipsilon* (Zhang et al., 2019), *Ostrinia nubilalis*, and *Plodia interpunctella* (Yue et al., 2003).

To validate findings in the laboratory, we conducted two field experiments in July and September of 2021, respectively. Our results showed that CHL + CLO and CYA + THI treatments resulted in control efficacy of 79.84%–87.88% at 7 DAE and 68.93%–77.94% at 14 DAE. These results were similar to the use of diamides alone and were consistent with laboratory results. Thus, CHL + CLO and CYA + THI treatments exhibited control efficacy equivalent to CHL and CYA treatment alone; however, it is important to mention that the dosage of diamides in the combined treatments was lower than the usage of CHL and CYA alone. These results suggest that synergistic action are present when the diaminde and neonicotinoid insecticides are simultaneously used. The snyergistic effect may be caused by the following two reasons: firstly, neonicotinoid insecticides block the metabolic systems of FAW that would break down diaminde molecules; secondly, neonicotinoid insecticides interfere with the detoxication of diamindes insecticides through their action on polysubstrate monooxygenases (PSMOs) and other enzyme systems (Bernard and Philogène, 1993). Most importantly, application of CHL + CLO and CYA + THI reduce the control cost when they are used to manage FAW larvae. For example, the control cost of CHL + CLO is 42.52 $ per hectare, which reduced 42.52 $ when compared to CHL (85.04 $ ha^−1^). Similarly, control cost of CYA + THI is 45.35 $ per hectare, which reduced 11.34 $ per hectare when compared to CYA (56.69 $ ha^−1^). Therefore, this strategy is helpful for large-scale application of these insecticides as seed treatments.

Moreover, the control efficacy of the four treatments in field experiments was lower than laboratory studies, which may be attributed to the rapid degradation of insecticide residues in field-grown corn. Weather conditions, application time, insecticide characteristics, and translocation within plants can influence the persistence of insecticides and may impact efficacy (Pfeil, 2014; Teló et al., 2015). Reduced control efficacy in the field can be impacted by: 1) environmental factors such as UV irradiation and rain (Lim et al., 1990; Lanka et al., 2014); 2) translocation to plant tissues where active ingredients may be diluted (Zhang et al., 2019); and 3) insecticide resistance. In addition, we observed that the control efficacy at 21 DAE or later was lower in summer months as compared to autumn. This may be caused by higher soil temperatures or elevated moisture levels during seedling emergence in summer, which may influence microbial activity and further contribute to insecticide degradation (Zhang et al., 2019).

To evaluate the effects of CHL + CLO and CYA + THI as seed treatments in corn, we measured growth indicators and evaluated residue concentrations and dynamics. Firstly, the emergence rates of corn seeds treated with CHL + CLO and CYA + THI were not different from other treatments and the untreated control both in laboratory and field experiments, which suggests that the combined insecticides had no adverse effects on emergence. Secondly, the CHL + CLO and CYA + THI treatments had stimulatory effects on measured parameters. Our results were different from those reported by Abdu-Allah and Hashem (2017) and Huang et al. (2015) but consistent with studies showing that neonicotinoid seed treatments could promote seed germination and increase primary root length, weight, and height of corn seedlings (Horii et al., 2007; Duan et al., 2012; Zhang et al., 2015). The stimulation of growth parameters may be the result of improved activity of antioxidants and stress-related enzymes such as guaiacol peroxidase and glucose-6-phosphate dehydrogenase (Ding et al., 2018). Previous studies have shown that insecticides, especially neonicotinoids, cause a decline in natural enemies and pollinators (Moser and Obrycki, 2009; Bredeson and Lundgren, 2018). Therefore, further studies are needed to explore the effects of CHL + CLO and CYA + THI on nontarget insects in the corn field. Finally, we observed that insecticide residues in the CHL + CLO and CYA + THI treatments gradually declined, and the half-life of the combined residues was relatively low when compared that of individual, single applications. Collectively, our results suggest that CHL + CLO and CYA + THI are relatively safe insecticides when applied to seeds and had no negative effect on corn growth.

## Conclusion

In summary, our results indicate that the application of CHL + CLO and CYA + THI as a corn seed treatment effectively controls FAW larvae on seedlings up to 14 DAE without compromising plant growth and development. Thus, CHL + CLO and CYA + THI are effective, environmentally-friendly insecticidal formulations that reduce the cost associated with single applications of CHL and CYA.

## Data Availability

The raw data supporting the conclusions of this article will be made available by the authors, without undue reservation.

## References

[B1] Abdu-Allah G. A. M. Hashem M. M. (2017). Efficiency and Side Effects of Three Neonicotinoid Insecticides Used as Faba Bean Seed Treatments for Controlling Cowpea Aphid. Egypt. Sci. J. Pestic. 3, 20–27. Available at: https://www.researchgate.net/publication/318701413 .

[B2] Azevedo R. D. Grutzmacher A. D. Loeck A. E. Silva F. F. D. Storch G. Herpich M. I. (2004). Effect of Seed Treatment and Leaf Spray of Insecticides in Different Water Volumes, on the Control of *Spodoptera frugiperda* (J.E. Smith, 1797) (Lepidoptera: Noctuidae), in Lowland Corn and Sorghum Crops. R.Bras. Agrociência. 10, 71–77. Available at: https://www.researchgate.net/publication/242633987 .

[B3] Bernard C. B. Philogène B. J. R. (1993). Insecticide Synergists: Role, Importance, and Perspectives. J. Toxicol. Environ. Health 38, 199–223. 10.1080/15287399309531712 8433403

[B4] Bolzan A. Padovez F. E. Nascimento A. R. Kaiser I. S. Lira E. C. Amaral F. S. (2019). Selection and Characterization of the Inheritance of Resistance ofSpodoptera frugiperda(Lepidoptera: Noctuidae) to Chlorantraniliprole and Cross‐resistance to Other Diamide Insecticides. Pest. Manag. Sci. 75, 2682–2689. 10.1002/ps.5376 30761724

[B5] Bredeson M. M. Lundgren J. G. (2018). Thiamethoxam Seed Treatments Reduce Foliar Predator and Pollinator Populations in Sunflowers ( Helianthus Annuus ), and Extra-floral Nectaries as a Route of Exposure for Seed Treatments to Affect the Predator, *Coleomegilla maculata* (Coleoptera: Coccinellidae). Crop Prot. 106, 86–92. 10.1016/j.cropro.2017.12.019

[B6] Carscallen G. E. Kher S. V. Evenden M. L. (2019). Efficacy of Chlorantraniliprole Seed Treatments against Armyworm (*Mythimna unipuncta* [Lepidoptera: Noctuidae]) Larvae on Corn (Zea mays). J. Econ. Entomol. 112, 188–195. 10.1093/jee/toy338 30388266

[B7] Ding J. Li H. Zhang Z. Lin J. Liu F. Mu W. (2018). Thiamethoxam, Clothianidin, and Imidacloprid Seed Treatments Effectively Control Thrips on Corn under Field Conditions. J. Insect Sci. 18 (6), 19. 10.1093/jisesa/iey128 30566643 PMC6299462

[B8] Duan Q. Zhao G. L. Jiang X. Y. Wang C. Bao J. Liu X. D. (2012). Effects of Imidacloprid Seed Dressing on the Seed Activity and the Seedling Growth of Maize. J. Maize Sci. 20, 63–69. 10.13597/j.cnki.maize.science.2012.06.015

[B9] Guo J. F. He K. L. Wang Z. Y. (2019). Biological Characteristics, Trend of Fall Armyworm *Spodoptera frugiperda*, and the Strategy for Management of the Pest. Chin. J. Appl. Entomol. 56, 361–369. 10.7679/j.issn.2095-1353.2019.045

[B10] Hannig G. T. Ziegler M. Marçon P. G. (2009). Feeding Cessation Effects of Chlorantraniliprole, a New Anthranilic Diamide Insecticide, in Comparison with Several Insecticides in Distinct Chemical Classes and Mode-Of-Action Groups. Pest. Manag. Sci. 65, 969–974. 10.1002/ps.1781 19449341

[B11] Horii A. Mccue P. Shetty K. (2007). Enhancement of Seed Vigour Following Insecticide and Phenolic Elicitor Treatment. Bioresour. Technol. 98, 623–632. 10.1016/j.biortech.2006.02.028 16581243

[B12] Huang L. Lu M. Han G. Du Y. Wang J. (2016). Sublethal Effects of Chlorantraniliprole on Development, Reproduction and Vitellogenin Gene (CsVg) Expression in the Rice Stem borer,Chilo Suppressalis. Pest. Manag. Sci. 72, 2280–2286. 10.1002/ps.4271 26939546

[B13] Huang L. Zhao C.-l. Huang F. Bai R.-e. Lü Y.-b. Yan F.-m. (2015). Effects of Imidacloprid and Thiamethoxam as Seed Treatments on the Early Seedling Characteristics and Aphid-Resistance of Oilseed Rape. J. Integr. Agric. 14, 2581–2589. 10.1016/S2095-3119(15)61140-6

[B14] Jiang Y. Y. Liu J. Wu Q. L. Ciren Z. G. Zeng J. (2021). Investigation on Winter Breeding and Overwintering Areas of *Spodoptera frugiperda* in China. Plant Prot. 47, 212–217. 10.16688/j.zwbh.2020432

[B15] Jiang Y. Y. Liu J. Xie M. C. Li Y. H. Yang J. J. Zhang M. L. (2019). Observation on Law of Diffusion Damage of *Spodoptera frugiperda* in China in 2019. Plant Prot. 6, 10–19. 10.16688/j.zwbh.2019539

[B16] Lahm G. P. Stevenson T. M. Selby T. P. Freudenberger J. H. Cordova D. Flexner L. (2007). Rynaxypyr: a New Insecticidal Anthranilic Diamide that Acts as a Potent and Selective Ryanodine Receptor Activator. Bioorg. Med. Chem. Lett. 17 (22), 6274–6279. 10.1016/j.bmcl.2007.09.012 17884492

[B17] Lanka S. K. Stout M. J. Beuzelin J. M. Ottea J. A. (2014). Activity of Chlorantraniliprole and Thiamethoxam Seed Treatments on Life Stages of the Rice Water Weevil as Affected by the Distribution of Insecticides in Rice Plants. Pest. Manag. Sci. 70, 338–344. 10.1002/ps.3570 23633166

[B18] Lim L. O. Scherer S. J. Shuler K. D. Toth J. P. (1990). Disposition of Cyromazine in Plants under Environmental Conditions. J. Agric. Food Chem. 38, 860–864. 10.1021/jf00093a057

[B19] Lutz A. L. Bertolaccini I. Scotta R. R. Curis M. C. Favaro M. A. Fernandez L. N. (2018). Lethal and Sublethal Effects of Chlorantraniliprole on *Spodoptera cosmioides* (Lepidoptera: Noctuidae). Pest. Manag. Sci. 74, 2817–2821. 10.1002/ps.5070 29766638

[B20] Martinelli S. Barata R. M. Zucchi M. I. DeCastroSilva-Filho M. Omoto C. Celso O. (2006). Molecular Variability of *Spodoptera frugiperda* (Lepidoptera: Noctuidae) Populations Associated to Maize and Cotton Crops in Brazil. J. Econ. Entomol. 99, 519–526. 10.1603/0022-0493-99.2.51910.1093/jee/99.2.519 16686155

[B21] Matsuda K. Ihara M. Sattelle D. B. (2020). Neonicotinoid Insecticides: Molecular Targets, Resistance, and Toxicity. Annu. Rev. Pharmacol. Toxicol. 60, 241–255. 10.1146/annurev-pharmtox-010818-021747 31914891

[B22] Moser S. E. Obrycki J. J. (2009). Non-target Effects of Neonicotinoid Seed Treatments; Mortality of Coccinellid Larvae Related to Zoophytophagy. Biol. Control 51, 487–492. 10.1016/j.biocontrol.2009.09.001

[B23] Muraro D. S. Stacke R. F. Cossa G. E. Godoy D. N. Garlet C. G. Valmorbida I. (2020). Performance of Seed Treatments Applied on Bt and Non-Bt Maize against Fall Armyworm (Lepidoptera: Noctuidae). Environ. Entomol. 49, 1137–1144. 10.1093/ee/nvaa088 32794557

[B24] Nault B. A. Taylor A. G. Urwiler M. Rabaey T. Hutchison W. D. (2004). Neonicotinoid Seed Treatments for Managing Potato Leafhopper Infestations in Snap Bean. Crop Prot. 23, 147–154. 10.1016/j.cropro.2003.08.002

[B25] Pes M. P. Melo A. A. Stacke R. S. Zanella R. Perini C. R. Silva F. M. A. (2020). Translocation of Chlorantraniliprole and Cyantraniliprole Applied to Corn as Seed Treatment and Foliar Spraying to Control *Spodoptera frugiperda* (Lepidoptera: Noctuidae). PLoS One 15, e0229151. 10.1371/journal.pone.0229151 32236101 PMC7112192

[B26] Pfeil R. (2014). Pesticide Residues: Pyrethroids. Encycl. Food Saf. 3, 31–34. 10.1016/b978-0-12-378612-8.00239-0

[B27] Qi G. J. Huang D. C. Wang L. Zhang Y. P. Xiao H. X. Shi Q. X. (2020). The Occurrence Characteristic in Winter and Year Round Breeding Region of the Fall Armyworm, *Spodoptera frugiperda* (J.E Smith) in Guangdong Province. J. Environ. Entomol. 42, 573–582. 10.3969/j.issn.1674-0858.2020.03.8

[B28] Ranabhat S. Wang C. (2020). Effect of Moisture on Efficacy of Selected Insecticide Dusts against the Common Bed Bug, *Cimex lectularius* (Hemiptera: Cimicidae). J. Environ. Entomol. 113, 1933–1939. 10.1093/jee/toaa122 32491179

[B29] Reisig D. D. Herbert D. A. Malone S. (2012). Impact of Neonicotinoid Seed Treatments on Thrips (Thysanoptera: Thripidae) and Soybean Yield in Virginia and North Carolina. Jnl. Econ. Entom. 105, 884–889. 10.1603/ec11429 22812126

[B30] Schemeer H. E. Bluett D. J. Meredith R. Heatherington P. J. (1990). “Field Evaluation of Imidacloprid as an Insecticidal Seed Treatment in Sugar Beet and Cereals with Particular Reference to Virus Vector Control,” in Proc Brighton Crop Prot Conf, Pest and Dis, BCPC, Alton, Hants, UK, 29–36. 0144-1612.

[B31] Selby T. P. Lahm G. P. Stevenson T. M. (2017). A Retrospective Look at Anthranilic Diamide Insecticides: Discovery and Lead Optimization to Chlorantraniliprole and Cyantraniliprole. Pest. Manag. Sci. 73, 658–665. 10.1002/ps.4308 27146435

[B32] Shiferaw B. Prasanna B. M. Hellin J. Bänziger M. (2011). Crops that Feed the World 6. Past Successes and Future Challenges to the Role Played by Maize in Global Food Security. Food Sec. 3, 307–327. 10.1007/s12571-011-0140-5

[B33] Sun X.-X. Hu C.-X. Jia H.-R. Wu Q.-L. Shen X.-J. Zhao S.-Y. (2021). Case Study on the First Immigration of Fall Armyworm, *Spodoptera frugiperda* Invading into China. J. Integr. Agric. 20, 664–672. 10.1016/s2095-3119(19)62839-x

[B34] Tang Q.-Y. Zhang C.-X. (2013). Data Processing System (DPS) Software with Experimental Design, Statistical Analysis and Data Mining Developed for Use in Entomological Research. Insect Sci. 20, 254–260. 10.1111/j.1744-7917.2012.01519.x 23955865

[B35] Taylor A. G. Eckenrode C. J. Straub R. W. (2001). Seed Coating Technologies and Treatments for Onion: Challenges and Progress. HortSci 36, 199–205. 10.21273/hortsci.36.2.199

[B36] Teló G. M. Senseman S. A. Marchesan E. Camargo E. R. Jones T. McCauley G. (2015). Residues of Thiamethoxam and Chlorantraniliprole in Rice Grain. J. Agric. Food Chem. 63, 2119–2126. 10.1021/jf5042504 25626153

[B37] Thrash B. Adamczyk J. J. Lorenz G. Scott A. W. Armstrong J. S. Pfannenstiel R. (2013). Laboratory Evaluations of Lepidopteran-Active Soybean Seed Treatments on Survivorship of Fall Armyworm (Lepidoptera: Noctuidae) Larvae. Fla. Entomol. 96, 724–728. 10.1653/024.096.0304

[B38] Wang S. Y. Zhu Q. Z. Tan Y. T. Ma Q. L. Wang R. F. Zhang M. F. (2019). Artificial Diets and Rearing Technique of *Spodoptera frugiperda* (J. E. Smith) in Laboratory. J. Environ. Entomol. 41, 742–747. 10.3969/j.issn.1674-0858.2019.04.8

[B39] Wang Y. Q. Ma Q. L. Tan Y. T. Zheng Q. Yan W. J. Yang S. (2019). The Toxicity and Field Efficacy of Chlorantraniliprole against *Spodoptera frugiperda* . J. Environ. Entomol. 42, 782–788. 10.3969/j.issn.1674-0858.2019.04.14

[B40] Wu C. Xiong T. Yin Y. Zhong G. Feng X. (2020). The Effect of Prevention and Control against *Spodoptera frugiperda* by Corn Seeds Pelleting. J. Environ. Entomol. 42, 1314–1321. 10.3969/j.issn.1674-0858.2020.06.4

[B41] Yue B. Wilde G. E. Arthur F. (2003). Evaluation of Thiamethoxam and Imidacloprid as Seed Treatments to Control European Corn Borer and Indianmeal Moth (Lepidoptera: Pyralidae) Larvae. J. Econ. Entomol. 96, 503–509. 10.1603/0022-0493-96.2.50310.1093/jee/96.2.503 14994821

[B42] Zhang L. Greenberg S. M. Zhang Y. Liu T.-X. (2011). Effectiveness of Thiamethoxam and Imidacloprid Seed Treatments against *Bemisia tabaci* (Hemiptera: Aleyrodidae) on Cotton. Pest. Manag. Sci. 67, 226–232. 10.1002/ps.2056 21077123

[B43] Zhang P. Zhang X. Zhao Y. Ren Y. Mu W. Liu F. (2015). Efficacy of Granular Applications of Clothianidin and Nitenpyram against Aphis Gossypii (Glover) and Apolygus Lucorum (Meyer-Dür) in Cotton Fields in China. Crop Prot. 78, 27–34. 10.1016/j.cropro.2015.08.012

[B44] Zhang P. Zhang X. Zhao Y. Wei Y. Mu W. Liu F. (2016a). Effects of Imidacloprid and Clothianidin Seed Treatments on Wheat Aphids and Their Natural Enemies on Winter Wheat. Pest. Manag. Sci. 72, 1141–1149. 10.1002/ps.4090 26248607

[B45] Zhang Z. Xu C. Ding J. Zhao Y. Lin J. Liu F. (2019). Cyantraniliprole Seed Treatment Efficiency against Agrotis Ipsilon (Lepidoptera: Noctuidae) and Residue Concentrations in Corn Plants and Soil. Pest. Manag. Sci. 75, 1464–1472. 10.1002/ps.5269 30450808

[B46] Zhang Z. Zhang X. Wang Y. Zhao Y. Lin J. Liu F. (2016b). Nitenpyram, Dinotefuran, and Thiamethoxam Used as Seed Treatments Act as Efficient Controls against *Aphis Gossypii via* High Residues in Cotton Leaves. J. Agric. Food Chem. 64, 9276–9285. 10.1021/acs.jafc.6b03430 27960287

[B47] Zheng C. G. Li G. H. Lu X. J. Tang L. Chen Q. J. (2006). Effects of Environmental Factors on Control Efficiency of SeNPV Pesticides. J. Environ. Entomol. 28, 66–70. 10.1001/j.issn.6155(2006)02-066-05

[B48] Zhou Z. X. Tang J. H. Lü B. Q. Lu H. (2020). Indoor Toxicity and Field Efficacy of Four Kinds of Insecticide against *Spodoptera frugiperda* in Hainan. Chin. J. Trop. Agric. 40, 6–12. 10.12008/j.issn.1009-2196.2020

