# Supplementary material for: Seed Treatment with Diamide and Neonicotinoid Mixtures for Controlling Fall Armyworm on Corn: Toxicity Evaluation, Effects on Plant Growth and Residuality
Source: Front Chem. 2022 Jun 8;10:925171. doi: 10.3389/fchem.2022.925171 (PMC9213745; doi:10.3389/fchem.2022.925171)
Supplement: Supplementary file 1 [file Table1.DOCX]

**Table S1** Multiple reaction monitoring (MRM) parameters for analysis of clothianidin, thiamethoxam, chlorantraniliprole, cyantraniliprole and J9Z38.

| Compound | Molecular formula | Selected ion | Precursor Ion (m/z) | Qualifier Ion (m/z) | S-Lens voltages (V) | Collision Energy (V) |
| --- | --- | --- | --- | --- | --- | --- |
| clothianidin | C_6_H_8_ClN_5_O_2_S | [M+H]^+^ | 249.960 | 131.978 | 53 | 14 |
|  |  |  |  | 169.093^*^ |  | 14 |
| thiamethoxam | C_8_H_10_ClN_5_O_3_S | [M+H]^+^ | 291.956 | 181.058 | 59 | 22 |
|  |  |  |  | 211.108^*^ |  | 13 |
| chlorantraniliprole | C_18_H_14_BrCl_2_N_5_O_2_ | [M+H]^+^ | 483.916 | 285.696 | 73 | 27 |
|  |  |  |  | 452.724^*^ |  | 19 |
| cyantraniliprole | C_19_H_14_BrClN_6_O_2_ | [M+H]^+^ | 475.080 | 286.002^*^ | 75 | 19 |
|  |  |  |  | 444.104 |  | 18 |
| J9Z38 | C_19_H_12_BrClN_6_O | [M+H]^+^ | 456.991 | 188.043 | 90 | 29 |
|  |  |  |  | 299.020^*^ |  | 37 |

^*^ Value represents quantitative ion transition.
