# Supplementary material for: Seed Treatment with Diamide and Neonicotinoid Mixtures for Controlling Fall Armyworm on Corn: Toxicity Evaluation, Effects on Plant Growth and Residuality
Source: Front Chem. 2022 Jun 8;10:925171. doi: 10.3389/fchem.2022.925171 (PMC9213745; doi:10.3389/fchem.2022.925171)
Supplement: Supplementary file 2 [file Table3.DOC]

| Table S3 The cost of CHL+CLO, CYA+THI, CHL and CYA when they are used to control FAW larvae*. | | | |
| --- | --- | --- | --- |
| Insecticides | Doseage（g ai.ha^-1^) | Cost（$ha^-1^） | Reduced cost（$ha^-1^） |
| CHL | 90 | 85.04 | - |
| CHL+CLO | 72 | 42.52 | 42.52 |
| CYA | 43.2 | 56.69 | - |
| CYA+THI | 72 | 45.35 | 11.34 |

* The cost of each insecticide was calculated based on its sale price in Chinese market.
