# Supplementary material for: Seed Treatment with Diamide and Neonicotinoid Mixtures for Controlling Fall Armyworm on Corn: Toxicity Evaluation, Effects on Plant Growth and Residuality
Source: Front Chem. 2022 Jun 8;10:925171. doi: 10.3389/fchem.2022.925171 (PMC9213745; doi:10.3389/fchem.2022.925171)
Supplement: Supplementary file 3 [file Table2.DOCX]

Table S2 The estimated half time (t_1/2_) of residues in CHL, CYA, CHL+CLO, CYA+THI, CLO and THI treatments

| Treatments | Insecticides | Regression | R^2^ | t_1/2_ (d) |  |
| --- | --- | --- | --- | --- | --- |
| CHL | CHL | C_t_= 1.1289 e^-0.1594t^ | 0.9603 | 4.35 |  |
| CYA | CYA | C_t_= 0.6707e^-0.1360t^ | 0.9035 | 3.64 |  |
|  | J9Z38(metabolite of CYA) | Ct= 0.0270 e^-0.0998t^ | 0.8887 | 6.95 | |
| CHL+CLO | CHL | Ct= 28.1029 e^-0.2994t^ | 0.9995 | 2.32 |  |
|  | CLO | C_t_= 0.7319 e^-0.3069t^ | 0.9963 | 2.26 |  |
| CYA+THI | CYA | C_t_= 3.0504 e^-0.2261t^ | 0.9652 | 3.07 |  |
|  | J9Z38 | C_t_= 0.0364 e^-0.0893t^ | 0.9754 | 7.76 |  |
|  | THI | C_t_= 9.1695 e^-0.3477t^ | 0.9997 | 1.99 |  |
|  | CLO(metabolite of THI) | C_t_= 1.4722 e^-0.2595t^ | 0.9945 | 2.67 |  |
| CLO | CLO | C_t_= 13.1299e^-0.3222t^ | 0.9999 | 2.15 |  |
| THI | THI | C_t_= 6.6313e^-0.3455t^ | 0.9999 | 2.01 |  |
|  | CLO | C_t_= 1.4387 e^-0.3009t^ | 0.9998 | 2.30 |  |
